# Investigating ripple pattern formation and damage profiles in Si and Ge induced by 100 keV Ar^+^ ion beam: a comparative study

**DOI:** 10.3762/bjnano.15.33

**Published:** 2024-04-05

**Authors:** Indra Sulania, Harpreet Sondhi, Tanuj Kumar, Sunil Ojha, G R Umapathy, Ambuj Mishra, Ambuj Tripathi, Richa Krishna, Devesh Kumar Avasthi, Yogendra Kumar Mishra

**Affiliations:** 1 Inter University Accelerator Centre, Aruna Asaf Ali Marg, New Delhi 110067, India; 2 SmartMaterials, Mads Clausen Institute, University of Southern Denmark, Alison 2 6400, Denmark; 3 Amity Institute of Nanotechnolgy, Amity University, Noida 201303, India; 4 Katholieke University, Faculty of Engineering Science, Leuven 3000, Belgium; 5 Department of Nanoscience and Materials, Central University of Jammu, Jammu 181143, India; 6 Centre for Interdisciplinary Research and Innovation, University of Petroleum and Energy Studies University, Dehradun 248007, India

**Keywords:** atomic force microscopy, ion beam, nanopatterns, radiation damage, Rutherford backscattering spectrometry, transmission electron microscopy

## Abstract

Desired modifications of surfaces at the nanoscale may be achieved using energetic ion beams. In the present work, a complete study of self-assembled ripple pattern fabrication on Si and Ge by 100 keV Ar^+^ ion beam bombardment is discussed. The irradiation was performed in the ion fluence range of ≈3 × 10^17^ to 9 × 10^17^ ions/cm^2^ and at an incident angle of θ ≈ 60° with respect to the surface normal. The investigation focuses on topographical studies of pattern formation using atomic force microscopy, and induced damage profiles inside Si and Ge by Rutherford backscattering spectrometry and transmission electron microscopy. The ripple wavelength was found to scale with ion fluence, and energetic ions created more defects inside Si as compared to that of Ge. Although earlier reports suggested that Ge is resistant to structural changes upon Ar^+^ ion irradiation, in the present case, a ripple pattern is observed on both Si and Ge. The irradiated Si and Ge targets clearly show visible damage peaks between channel numbers (1000–1100) for Si and (1500–1600) for Ge. The clustering of defects leads to a subsequent increase of the damage peak in irradiated samples (for an ion fluence of ≈9 × 10^17^ ions/cm^2^) compared to that in unirradiated samples.

## Introduction

Scientific research varying from electronics to photonics, homeland security, high-resolution parallel patterning of magnetic media, biotechnology, and medicine are based upon nanotechnology. These applications require nanopatterning techniques to fabricate devices or structures. Although these structures may not be visible to the naked eye, they certainly have a visible impact on the mentioned applications. Nanopatterning is a very delicate procedure that is only possible with special techniques such as ion beam sputtering (IBS), with which one can achieve nanostructures in a controlled manner on a wide variety of substrates with required dimensions. There are reports from 1960’s, by Cunningham et al. [1] and Navez et al. [2], on the production of submicron and nanoscale patterns by IBS. However, with the availability of high-resolution tools such as atomic force microscopy (AFM) [3] and transmission electron microscopy (TEM), it is possible to visualize these features. Formation of dots, ripples, and pits have been well studied using IBS [4–9]. In the last few decades, numerous efforts have been made to understand IBS through simulations [10] as well as experimental results [11–12]. Thanks to these efforts, desired nanopattern features with a large degree of control may be achieved, according to specific applications, on a wide variety of targets. Facsko et al. [13] have shown controlled growth of nanodots on GaSb, and their probable use was demonstrated via photoluminescence spectroscopy. Stupp et al. [14] have explored possible applications of self-assembly of biomolecules with controlled stereochemistry in materials technology. However, the fundamental reasoning behind how this self-organization process evolves in terms of defect creation or damage still needs to be understood.

Despite controlled fabrication of patterns has been achieved, details that influence the process of self-assembly still remain open. Ion beam sputtering is an important method for inducing topographical changes in specific materials. For silicon, self-organized dots, ripples, and cones have been observed [15], which may be achieved by altering the ion incidence angle [16]. However, there are some inconsistencies and replicability issues between the studies, which might mean that other experimental parameters might be important for the formation of these nanostructures. Computational studies have provided relevance and connections between experiments and theoretical modelling [4,17–24]. Basic models to explain IBS were initially given by Thompson et al. [25] and Sigmund et al. [26], based on the radiation damage in bulk materials. When an energetic ion strikes a target surface, it may lose its energy in the following ways. If the ion has enough energy to cross the repulsive potential energy barrier of target atoms at the surface, it will pass through the solid. A collision cascade is created within the target atoms during the slowing-down course. The impinging ion subsequently transfers its energy to the atoms of the target material in all the collisions and finally stops. When this energy transfer is sufficient, a displacement of atoms from their equilibrium positions creating a vacancy or a recoil occurs. Alternatively, if the ion energy is high enough such recoils may create additional displacement of atoms through a cascade effect with subsequent decrement in their energy. In due course, the recoil energies are quenched in short timescales of ≈10^−15^ seconds. The modified target volume, which gets affected due to this slowing down process, depends on the mass and energy of the incoming ion and on the mass of the target atom. It may be expressed as the spatial distribution of the energy transferred/deposited within the target [27–28]. Sometimes the energy distribution on the target atoms at the surface may be sufficient to overcome binding energies so as to knock them out of the surfaces through an outwardly directed momentum. This process is known as sputtering [26], and the number of ejected atoms per ion is given by the sputtering yield, *Y*(θ). It is clearly visible that *Y* is a function of the incident angle θ, and it maximizes around θ ≈ 60°. Ion–matter interaction in low-energy regimes is well understood; however, a few empirical additions have been taking place in the formulism based upon the experimental observations [29–30]. A large group of theoreticians have contributed to the already existing classic description given by Bradley and Harper [31] based on morphological effects of IBS. The height *h*(*x*, *y*, *t*) of the sputtered surface can be described by a linear equation (Equation 1):


[1]

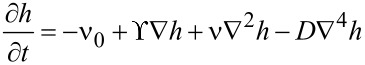



where ν_0_ is the constant erosion velocity, ν is the effective surface tension, and *D* denotes the surface diffusion which is activated by different physical processes (i.e., thermal diffusion and ion-induced diffusion) [32]. This approach is based on the linear cascade model and Gaussian approximation of energy distribution as developed by Sigmund [26] to describe ion–atom collisions inside the target.

Rutherford backscattering spectrometry (RBS) studies in the channelling mode have been used to study defects in crystals for more than a few decades now [33–34]. It imparts the useful information about the structure and composition of materials through the damage fraction studies of ion-bombarded crystalline samples by detecting the backscattered beam of high-energy ions (He^+^ ≈1–2 MeV). It impinges on the target material which provides good mass and depth resolution and also probes smaller radiation damages [35]. The damage produced by ion implantation in semiconductors consists of randomly distributed atoms displaced from their regular lattice sites up to a depth or range of damage profiles. Single-crystal materials (e.g. silicon and germanium) are composed of ordered arrays of atoms. If an ion beam is aligned to the atomic planes, most of the ions pass through the interplanar space and penetrate deep into the crystal. This can be used in channelling studies to analyse the crystal structure and to locate interstitial atoms within the array of target atoms. The relation between yield and defect concentration was derived by Bøgh [33]. It provides information about the depth distribution of defects in the first few microns beneath the crystalline surface. Channelling is an important process which has been heavily studied by Norlund et al. [24] and Hobler et al. [36]. Thus, in our studies, RBS-c plays a significant role in understanding the damage fractions in Si and Ge due to Ar^+^ ions. In the present work, 100 keV Ar^+^ ion bombardment was simultaneously performed on Si and Ge substrates. A correlation between ripple morphology and ion beam parameters was derived to understand the damage incurred by Ar^+^ ions inside both materials using RBS-c measurements.

## Experimental Details

Commercially available Si and Ge wafers, procured from Semiconductor wafers Inc., were taken and cut into equal pieces of 0.5 cm × 1 cm. The samples were mounted on an experimental ladder used for irradiation experiments using double-sided tape. Pieces of Si were mounted on the left-hand side and of Ge on the right-hand side of the Cu ladder. This was done to allow them to be simultaneously irradiated, keeping the experimental conditions the same. The experiment was performed at high vacuum ≈5 × 10^−6^ Torr and at room temperature. Argon ions (100 keV) have been used to irradiate the samples at an incident angle of θ ≈ 60° with respect to the surface normal [16]. The area in which the ion beam fell was kept larger (1 × 1) cm^2^ to allow for the simultaneous irradiation of both samples (Si and Ge). The time was calculated using Equation 2:


[2] $$T\left(\sec\right)=\left(\phi \times A\right)/\left(I\times 6.25\times 10^{9}\right),$$

where ϕ is the ion fluence in ions/cm^2^, *A* is the area of the sample in cm^2^, and *I* is the current in particle per nanoampere.

The ion fluence was chosen from the literature [37–38] as 3, 5, 7, and 9 × 10^17^ ions/cm^2^ to induce complete amorphization within the two surfaces up to the ion range. The ion irradiation experiment was performed in the 90-degree beam line dedicated for materials science experiments in the Low-Energy Ion Beam (LEIB) facility of the Inter University Accelerator Centre, New Delhi. The electronic and nuclear energy losses of 100 keV Ar^+^ inside Si and Ge were calculated using the SRIM software [39]. The electronic energy loss values were found to be 37.67 and 36.51 eV/Å for Si and Ge, respectively, and the nuclear energy loss values were found to be 47.75 and 59.61 eV/Å for Si and Ge, respectively. The range of Ar ions in Si is 106.5 nm and that in Ge is 72.2 nm. The pristine and irradiated samples were characterized by AFM (Nanoscope IIIa controller, Bruker, USA) using an RTESP tip with radius of curvature of ≈10 nm. The RBS measurements were performed in channelling mode using 2 MeV He^+^ ions at the PARAS facility at the Inter University Accelerator Centre (IUAC), New Delhi, and transmission electron microscopy (TEM) using an equipment from Jeol, Japan. The samples for TEM were prepared for cross-sectional studies in the TEM sample preparation lab at IUAC, New Delhi.

## Results and Discussion

### Atomic force microscopy studies

**Figure 1:**
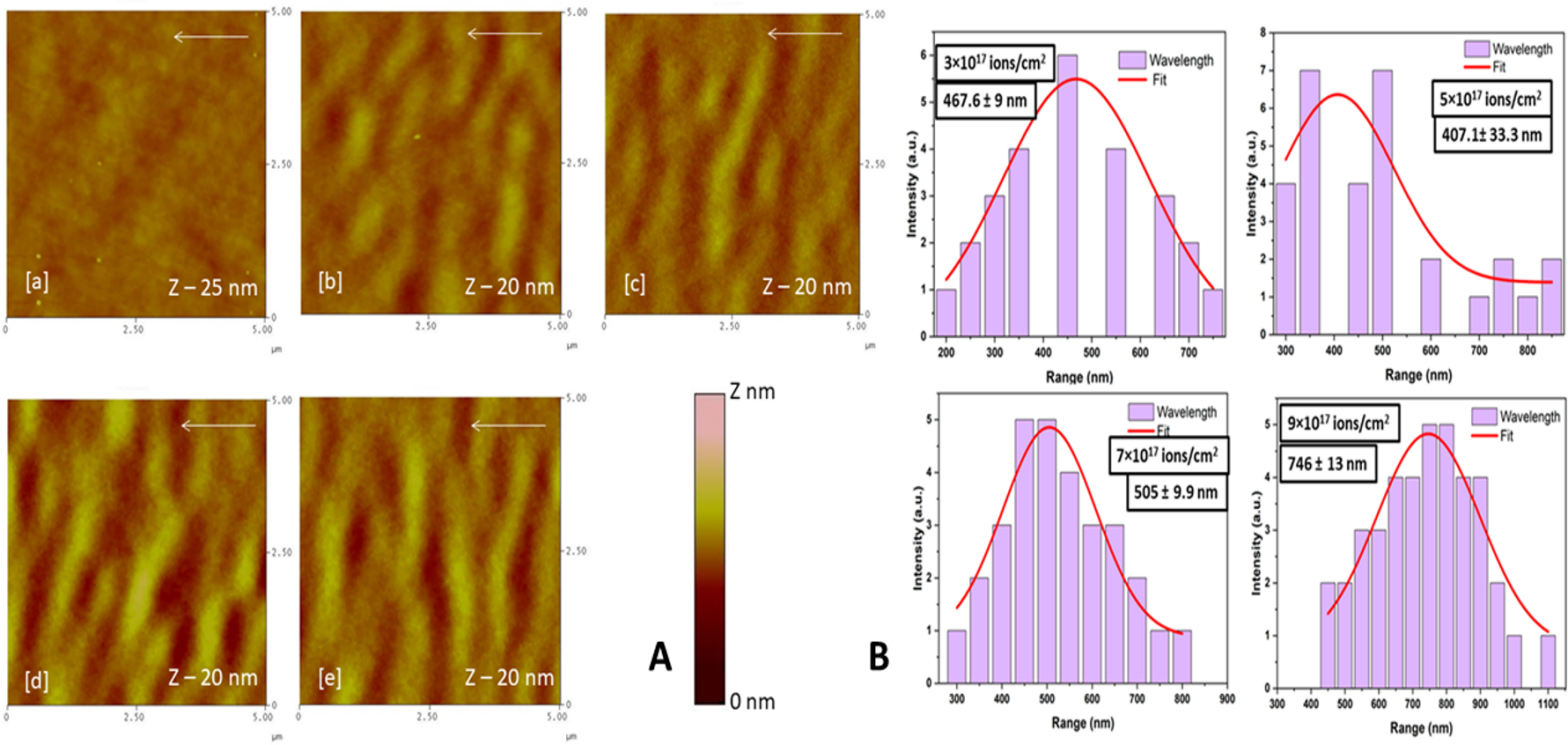
A. AFM images of pristine and 100 keV Ar^+^ ion-irradiated Si samples. (a) Pristine and irradiated samples with (b) 3 × 10^17^, (c) 5 × 10^17^, (d) 7 × 10^17^, and (e) 9 × 10^17^ ions/cm^2^ (all images are in 5 µm × 5 µm scan size) with corresponding wavelength distribution shown in B.

Energetic ions, of a few hundreds of kiloelectronvolts, from the ion implanters modify the surface of the target material to grow nanopatterns. The surfaces of the pristine and ion-treated samples were studied via AFM for the surface topography and change in root-mean-square (RMS) surface roughness. Figure 1 shows AFM images of pristine and 100 keV Ar^+^ ion-irradiated Si samples. Pristine samples show a smooth surface with a surface roughness of ≈0.5 nm as observed in Figure 1A (a).

**Figure 2:**
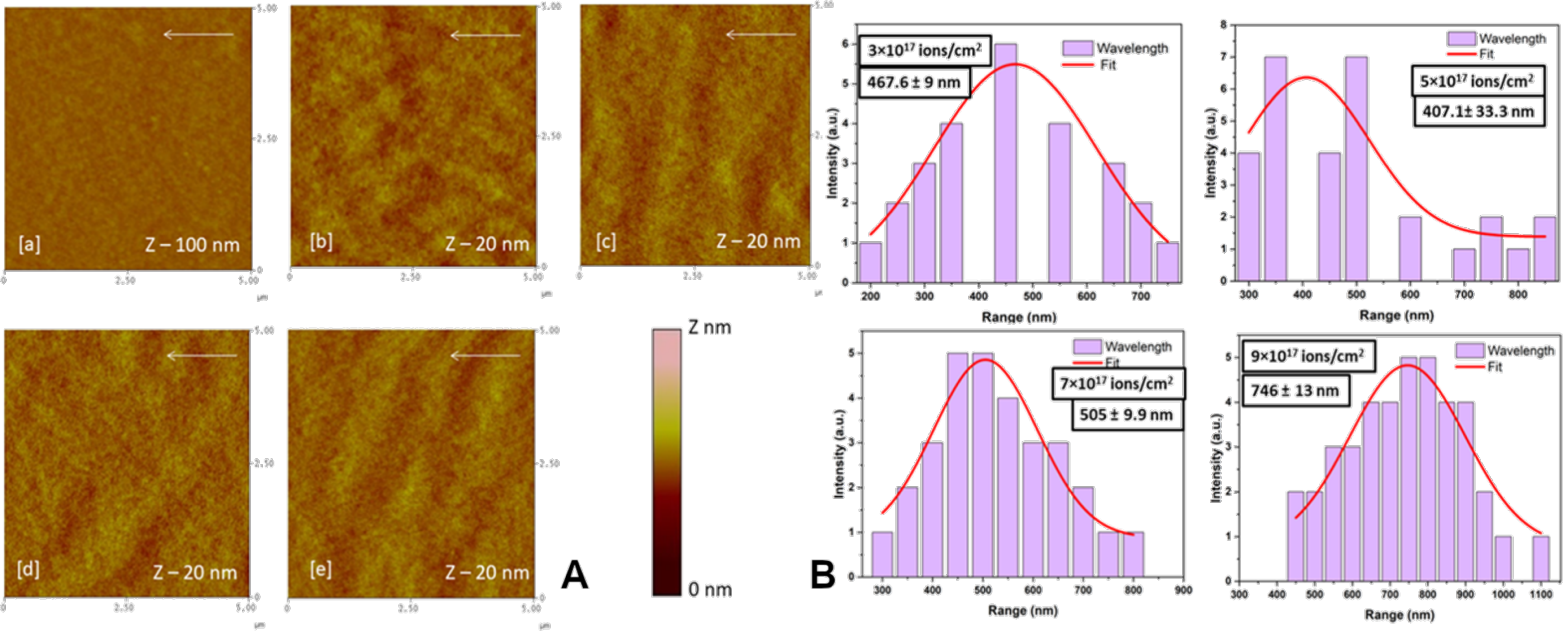
A: AFM images of pristine and 100 keV Ar^+^ ion-irradiated Ge samples (a) pristine, (b) 3 × 10^17^, (c) 5 × 10^17^ (d) 7 × 10^17^ and (e) 9 × 10^17^ ions/cm^2^ irradiated samples (all images are in 5 µm × 5 µm scan size), B shows the corresponding wavelength distribution.

Figure 1A (b–e) shows the surface topography of the irradiated samples at respective ion fluences of (b) 3 × 10^17^, (c) 5 × 10^17^, (d) 7 × 10^17^, and (e) 9 × 10^17^ ions/cm^2^. The surface roughness (*R*_q_) is found to be increased with ion fluence from ≈1.0 nm to 1.6 nm due to ion-induced sputtering at a 60° incidence angle. The pattern formation starts on any surface with amorphization through ion-induced defects resulting from collision cascades [16]. As shown in the AFM micrographs, the Si surface shows ripple patterns. These ripples are more organized and become more regular with ion fluence, with a ripple wavelength ranging from 400 to 740 nm (Figure 1B). The ripple patterns are more pronounced and have a preferential orientation perpendicular to the ion beam direction (shown by an arrow on each of the AFM images). The wavelength of the ripples obtained for initial fluences was ≈430 nm, and became wider (≈740 nm) for an ion fluence of 9 × 10^17^ ions/cm^2^. Figure 2A shows AFM images of pristine and 100 keV Ar^+^ ion-irradiated Ge samples. The pristine sample shows a smooth surface with roughness of ≈0.5 nm as observed in Figure 2A (a). Figure 2A (b–e) shows AFM images of irradiated samples at respective ion fluences of (b) 3 × 10^17^, (c) 5 × 10^17^, (d) 7 × 10^17^, and (e) 9 × 10^17^ ions/cm^2^. The Ge surface shows a slight change in the surface roughness to 0.6 nm when irradiated with an ion fluence of 3 × 10^17^ ions/cm^2^. At a fluence of 5 × 10^17^ ions/cm^2^, ripple patterns start appearing on the surface perpendicular to the beam direction (shown by the white arrow in Figure 2) with a roughness of 0.65 nm.

The ripples have an average wavelength of ≈370 nm as shown in Figure 2B, with wavelength plots obtained for various ion fluences in the case of Ge samples after irradiation. The wavelength of the ripples obtained for initial fluences was ≈350 nm. The RMS surface roughness of both Si and Ge samples increases with ion fluence. For Si, it changes from 0.5 to 1.2 nm and for Ge it changes from 0.5 to 0.65 nm. It is observed that the patterns formed on Si are more prominent than those on Ge, and this may be due to the choice of Ar^+^ ions. It has been shown in the literature that the probability of formation of ripples with Ar^+^ ions is higher for Si (*m* = 28 amu) than that for Ge (*m* = 72 amu) [9]. This may be due to the mass difference between Si and Ar^+^ (*m* = 40 amu), which is smaller compared to that between Ar^+^ and Ge. Therefore, when Ar^+^ ions enter the surface of Si, it amorphizes the near surface by inducing defects and irregularity in the Si crystal. On the other hand, since Ge is more massive, it probably requires an ion more massive than Ar or with more energy, ion fluence, or elevated temperature to induce that type of energy deposition in the Ge lattice. This way, the defects can be produced and the substrate can be amorphized. The roughness and growth exponents have been deduced from the RMS surface roughness and power spectral density data to understand the mechanism of ripple formation on Si and Ge. From the slope, *n*, of the linear part of power spectral density (PSD) curves, one may find the roughness parameter α using the formula α = (*n* − 1)/*d*, where *d* is the dimension of PSD [40]. In our case it is 2. Further, from the log plot of RMS roughness as a function of ion fluence, the slope is obtained as 0.23 ± 0.07 and 0.19 ± 0.09 for Si and Ge, respectively. The roughness parameter α and the growth parameter β give information about the mechanism responsible for creating these surface structures due to the interplay between roughness induced by sputtering and smoothening due to diffusion processes. The values for α and β were found to be α = 0.42 and 0.26 and β = 0.23 and 0.19 for Si and Ge, respectively, indicating that sputtering dominates in both cases to create ripples on the two surfaces. However, this process is better for Si.

### Transmission electron microscopy studies

The TEM analysis of Si and Ge samples irradiated with a fluence of 9 × 10^17^ ions/cm^2^ was performed in cross-sectional mode. The TEM image clearly reveals the surface modification occurred due to Ar ion irradiation.

**Figure 3:**
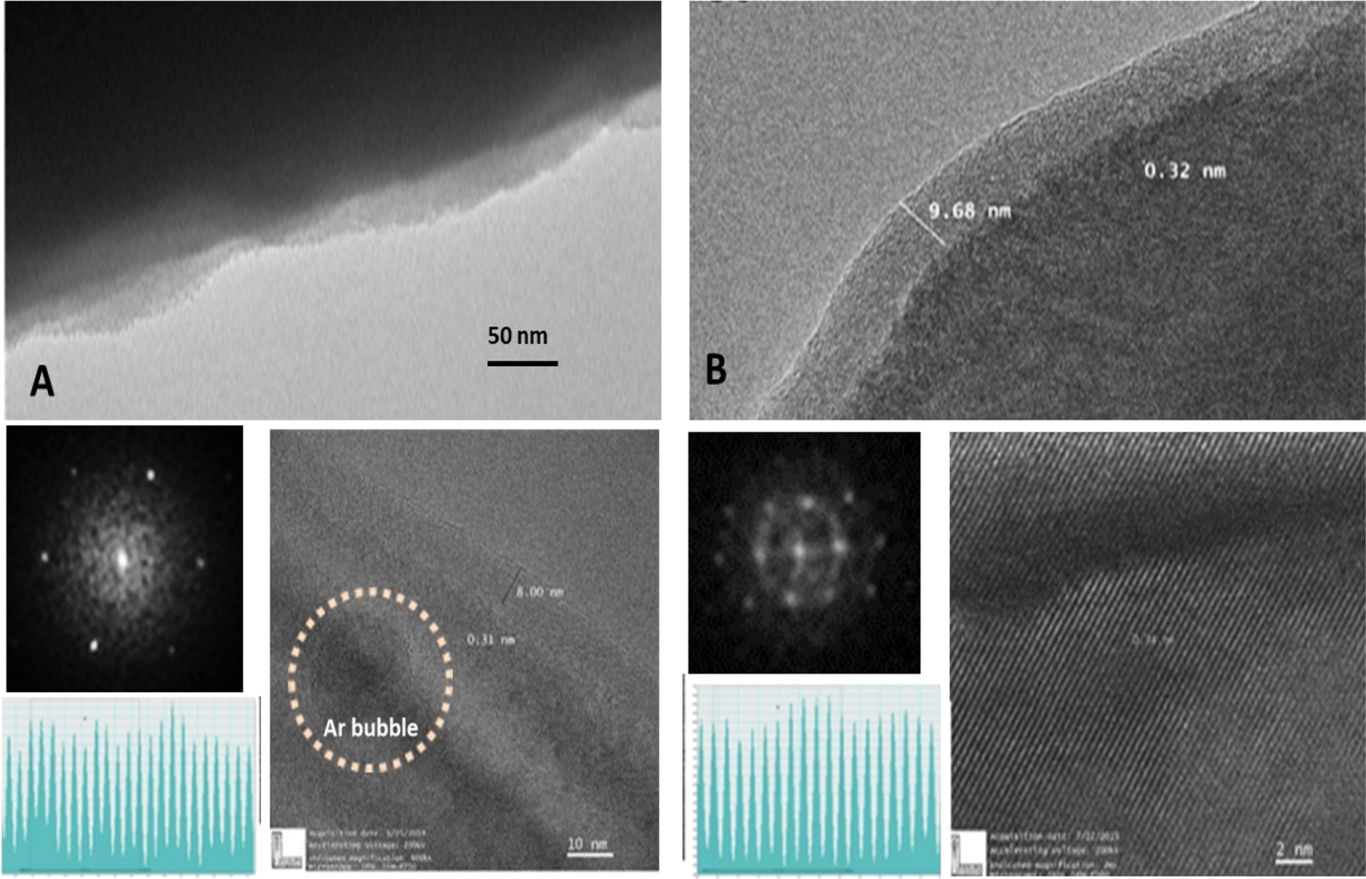
TEM images of A. Si and B. Ge samples irradiated with 9 × 10^17^ ions/cm^2^, with corresponding SAED patterns.

At fewer places, Ar bubbles of ≈15 nm were also visible (marked with a dotted section) in Si. It was found that the samples were still crystalline as seen in Figure 3A and Figure 3B for Si and Ge, respectively with corresponding *d*-spacing and selected area electron diffraction (SAED) pattern. It was observed that Ge exhibits higher crystallinity as compared to that of Si, even after irradiation at 9 × 10^17^ ions/cm^2^. Due to a higher mass difference between Si and Ge, Ge being a heavier target, there was not too much damage as a result of Ar ion irradiation. Therefore, most of the region remained unaltered beyond the ion penetration depths for both cases. This may be due to the high penetration depth of the electron beam rather than the range of the ion beam. The *d*-spacing found for Si was 0.31 nm and for Ge was 0.34 nm. The SAED pattern clearly shows a good diffraction pattern for Ge indicating that crystallinity remained intact for Ge even after irradiation at a higher ion fluence than that observed for Si. This further confirms that the near surface region of Si, amorphized to form better ripple patterns in Si as compared to Ge, may be due to the overlap of the collision cascade which led to more defect formation [41].

### Rutherford backscattering results

**Figure 4:**
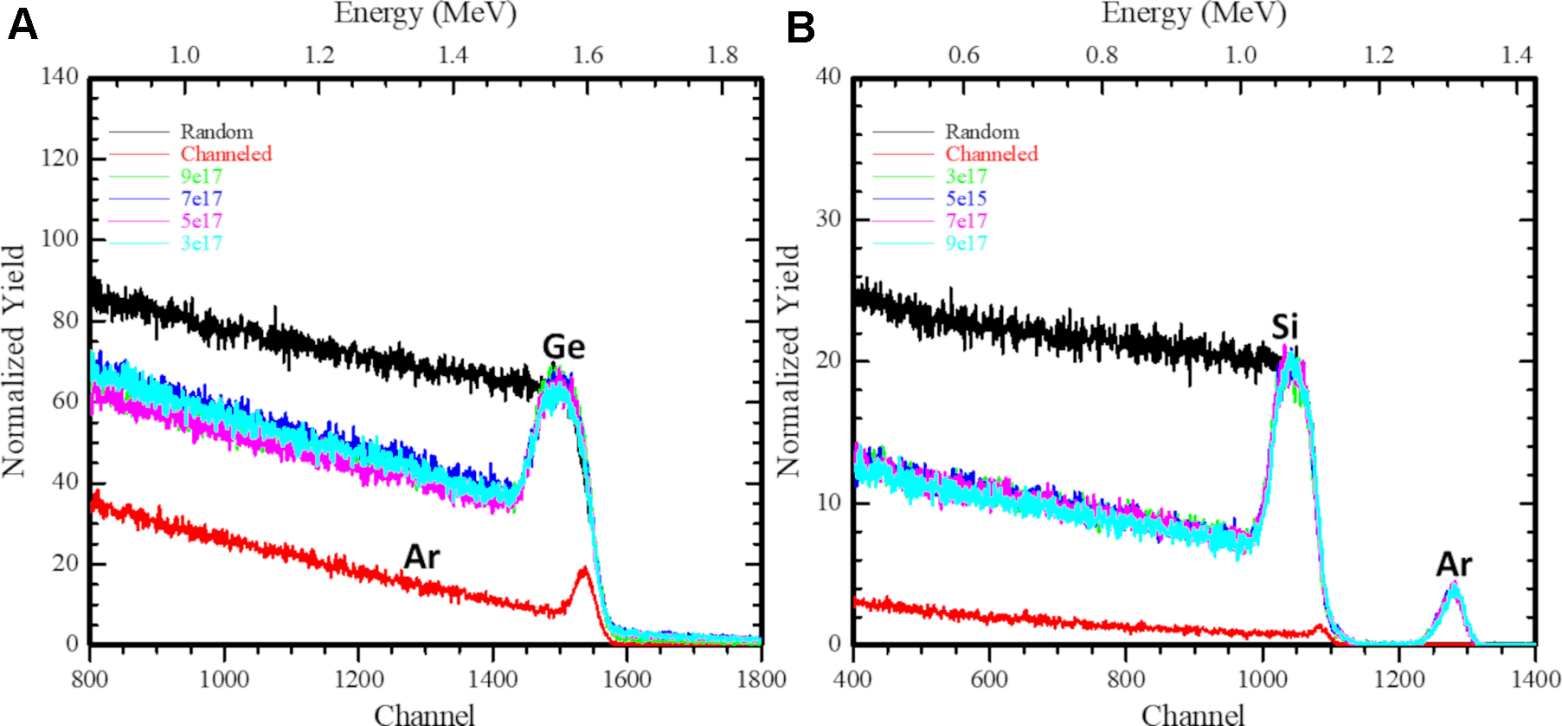
Aligned spectra for (A) Ge and (B) Si targets before and after ion irradiation with 100 keV Ar^+^ ions at ion fluences varying from 3 to 9 × 10^17^ ions/cm^2^.

The RBS technique was used to determine the structure and composition of the materials by measuring the backscattering of a beam of high-energy ions (He^+^) impinging on a sample. The RBS-channelling spectra of deep ion implants in Si and Ge were analysed to extract the depth profiles of the displaced atoms. Figure 4 shows the aligned spectra for Ge(111) and Si(111) targets before and after ion irradiation with 100 keV Ar^+^ ions at various ion fluences from 3 to 9 × 10^17^ ions/cm^2^. The crystallinity of the investigated target was determined by the comparison of the aligned spectrum with the random spectra (black). For the pristine Si and Ge samples, the backscattered (BS) yield in an aligned direction reduces to 5% and 7%, respectively. In defect analysis through ion implantation [42], the category-I damage is the subthreshold damage (i.e., partially damaged region) before it completely turns amorphous. On a complete amorphization, an amorphous/crystalline (a/c) interface is formed and further incoming ions create damage beyond this interface or end-range (ER) defects are produced. The irradiated Ge and Si targets clearly show visible damage peaks between channel numbers (1000–1100) for Si and (1500–1600) for Ge. The clustering of defects leads to the subsequent increase of the damage peak in irradiated samples (for an ion fluence of ≈9 × 10^17^ ions/cm^2^) compared to that of unirradiated samples. Typically, an obtained *Χ*_min_ value of 4 to 5% indicates good quality of Si and Ge pristine crystals. The increase of de-channelling is attributed to the accumulation of defects produced by Ar irradiation.

**Figure 5:**
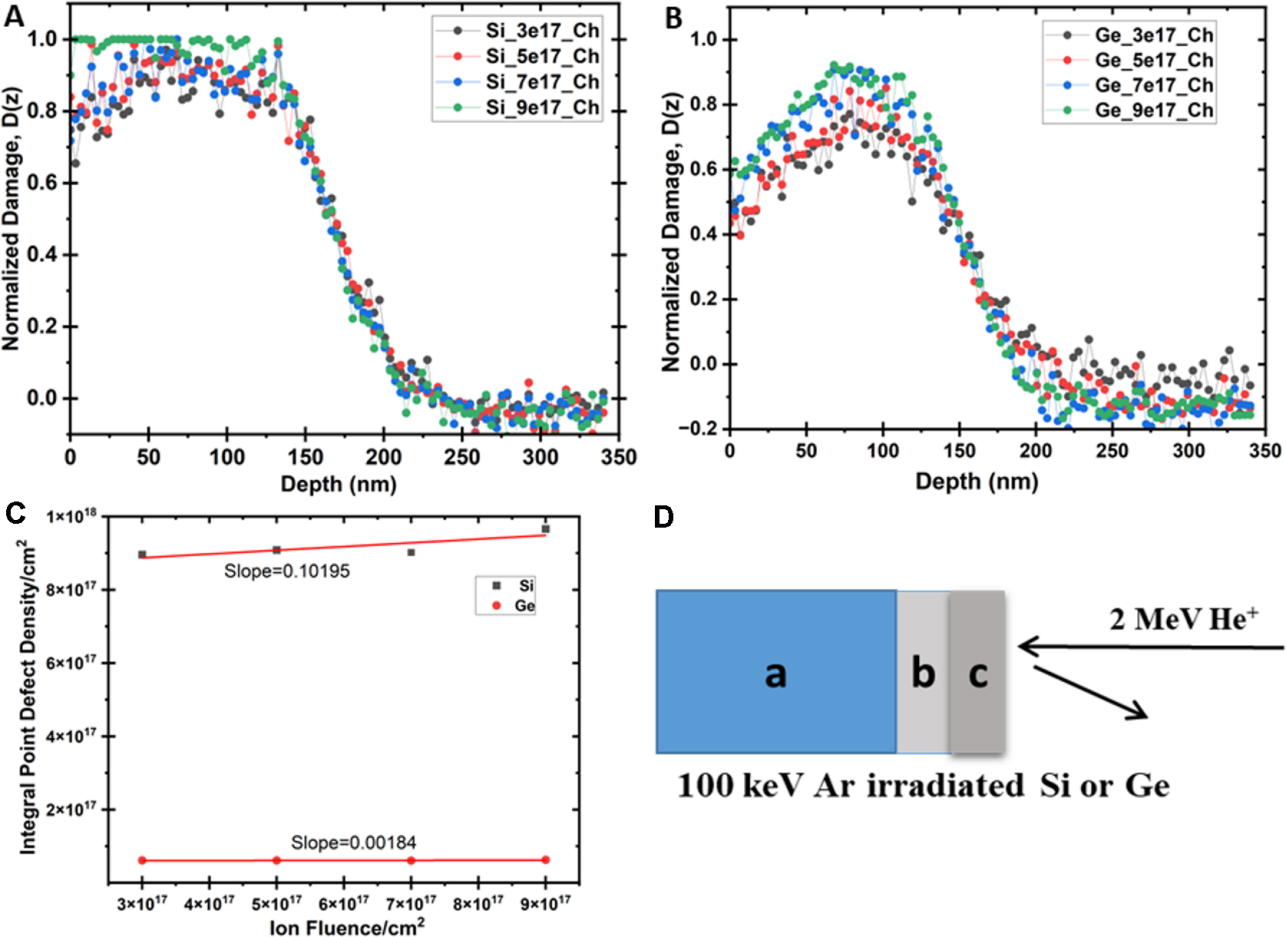
Damage fractions calculated for (A) Si and (B) Ge after irradiation (using the DICADA code. (C) Density of point defects with an increase in ion fluence. (D) Schematic representation of the analysis of amorphous (c) and amorphous/crystalline (b) zones with RBS-c.

The RBS-c spectra recorded for Si and Ge single-crystal samples pre-damaged with 100 keV Ar^+^ ions at RT are presented in Figure 5A and Figure 5B, respectively. The RBS spectrum recorded for the pristine sample in random orientation displays two steps corresponding to the backscattering from Si and Ar for channel numbers ≈1050 and ≈1275, respectively. Defect clustering may lead to a subsequent increase of the damage peak in irradiated samples as compared to that of pristine Si and Ge samples. To understand the evolution of the lattice disorder with ion irradiation conditions, the RBS-c data were analyzed and the damage depth distribution (i.e., finite-difference frequency-domain, FDFD, as a function of depth) was extracted by using the simulation code “De-channelling In Crystals And Defect Analysis (DICADA)” [35]. The RBS-c results show Ar in Si spectra, however, due to a lower mass, it is not observed in Ge spectra. The Ar^+^ estimated fluence matches the implanted fluence ruling out the possibility of sputtering for Ge. However, Si shows the damage peak around 80 nm with some sputtering of Si atoms. Ar is a period III element, thus, it generates a lower stress field. The depth distribution of defects for pre-damaged crystals before and after Ar^+^ irradiation is shown in Figure 5 for Si and Ge. As the Ar^+^ fluence is increased, the damage level increases by different amounts towards both the crystal surface up to the depths of 158 nm (A) and 170 nm (B) with an integral density of point defects of ≈0.17 × 10^18^ per cm^2^ and 0.38 × 10^17^ per cm^2^ for Ge and Si, respectively, as calculated by DICADA. This may be due to the tailing effects where an incoming ion comes to rest after travelling a certain distance inside the target material, taking into account the ion straggling.

For Si and Ge, a damage peak is exhibited around ≈75 nm , and the damaged layer extends up to a depth of ≈110 nm, which is consistent with the range of ions calculated with the SRIM code [39,43]. Here, the low-energy part of the spectrum continuously increases, which shows that both samples comprised of in-depth defects even before irradiation, and these defects are high in number in the case of Si (Figure 5C) in comparison to Ge. The area of the damage peak indicates that there are defect accumulations in the irradiated region as shown in Figure 5C, which confirms the near surface amorphization in Si in comparison to Ge.

**Figure 6:**
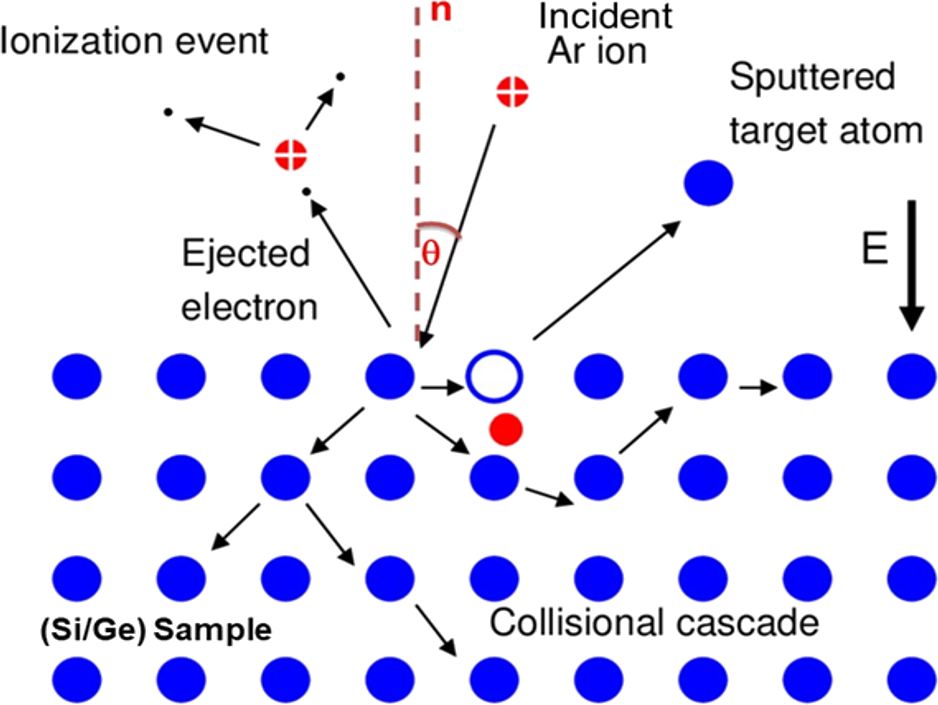
Schematic representation of various defects produced due to low-energy ion interaction with target atoms.

The main defects are the point defects such as interstitials and vacancies produced due to energy transfer between the incoming ions and the target atoms (Figure 6). The range of defect depths calculated from RBS-c spectra for Ar^+^ ions irradiated on Ge is 3 to 4% which is lower than that for Si, having 10 to 11% due to differences in mass values of the crystal atoms. However, the damage distribution within the amorphous layer is greater for Ge in comparison with Si implanted samples, and it increases with ion fluence.

## Conclusion

The RBS-c is an effective characterization technique for the estimation of amorphous depth and defect concentration in implanted single crystals. The RBS-c analysis for point defects shows a linear behaviour in defect density with ion fluence. The formation of highly dense dislocation loops beyond the a/c interface results in an increase in the BS yield towards lower channel numbers. The RBS is effective and accurate in determining the sputtering yield for Si and Ge upon incident Ar^+^ at 60°. It can be concluded that Ar is creating more defects in Si as compared to that in Ge, resulting in near surface amorphization. Thus, it promotes ripple formation as observed from AFM images. The ripple wavelength increases with ion fluence in both cases. We observed better ripples in Si in comparison to those in Ge, and the Ge surface shows shallow ripples at fewer spots which were irregular, thus, the ripple formation on Ge is also confirmed.

## Supporting Information

File 1 Supplementary files.

## Data Availability

The data that supports the findings of this study is available from the corresponding author upon reasonable request.
