# Supplementary material for: Investigating ripple pattern formation and damage profiles in Si and Ge induced by 100 keV Ar+ ion beam: a comparative study
Source: Beilstein J Nanotechnol. 2024 Apr 5;15:367–75. doi: 10.3762/bjnano.15.33 (PMC10999981; doi:10.3762/bjnano.15.33)

BEILSTEIN JOURNAL OF NANOTECHNOLOGY

Supporting Information

for

Investigating ripple pattern formation and damage profiles in Si and Ge induced by 100 keV Ar^+ ion beam: a comparative study

Indra Sulania, Harpreet Sondhi, Tanuj Kumar, Sunil Ojha, G R Umapathy, Ambuj Mishra, Ambuj Tripathi, Richa Krishna, Devesh Kumar Avasthi and Yogendra Kumar Mishra

Beilstein J. Nanotechnol. **2024,** *15,* 367–375. doi:10.3762/bjnano.15.33

Supplementary files

License and Terms: This is a supporting information file under the terms of the Creative Commons Attribution License (https://creativecommons.org/licenses/by/4.0). Please note that the reuse, redistribution and reproduction in particular requires that the author(s) and source are credited and that individual graphics may be subject to special legal provisions.
The license is subject to the *Beilstein Journal of Nanotechnology* terms and conditions: (https://www.beilstein-journals.org/bjnano/terms)

1. Si samples after irradiation at 10×10 µm^2

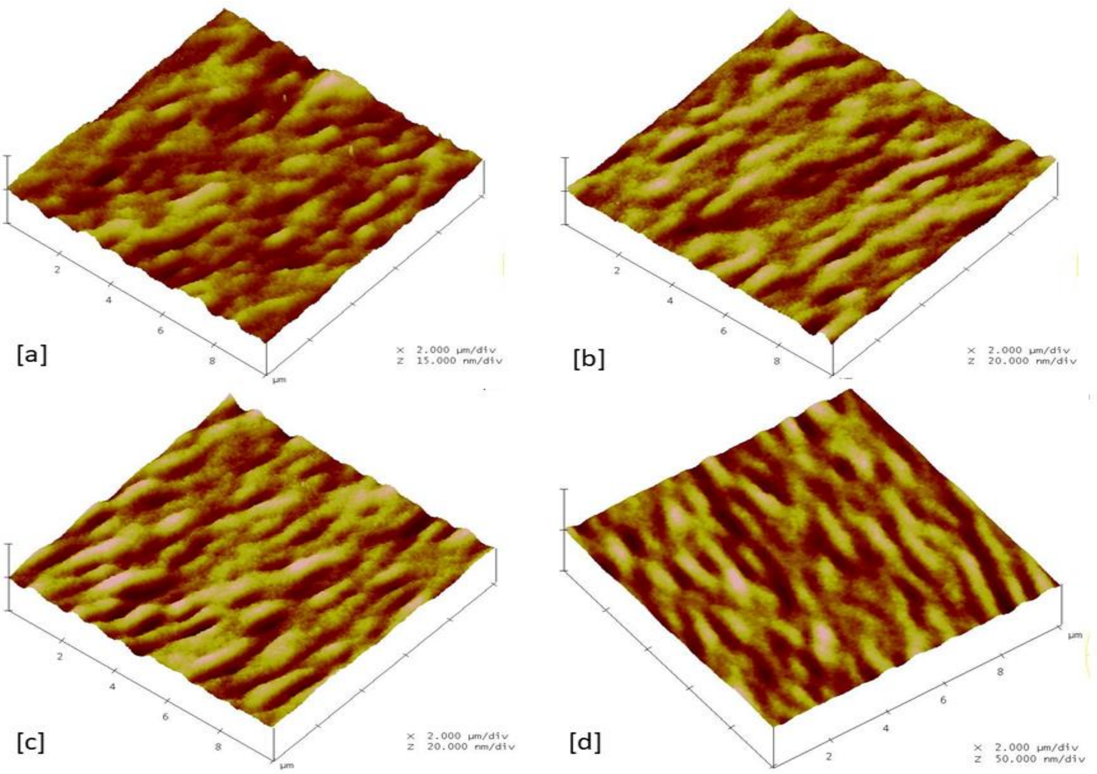

2. Ge samples after irradiation at 10×10 µm^2

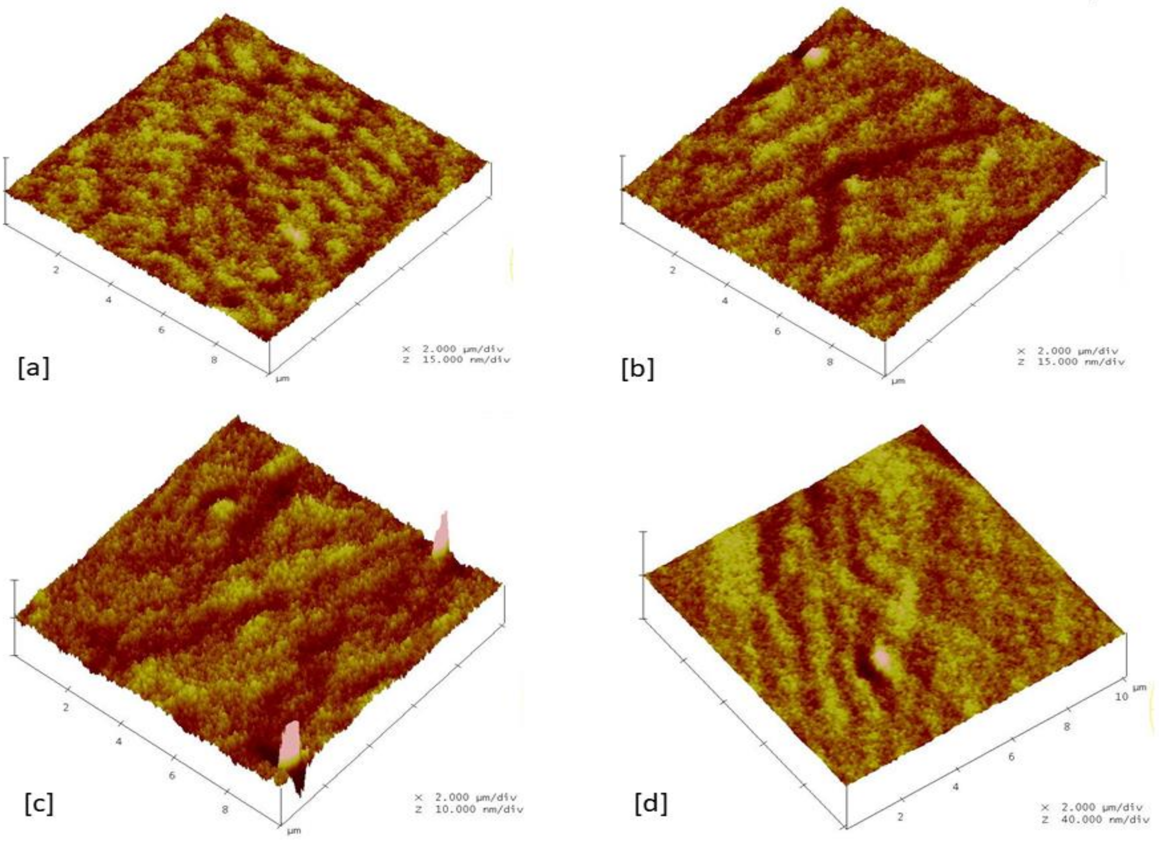

Supplement: File 1 — Supplementary files. [file Beilstein_J_Nanotechnol-15-367-s001.pdf]
